# Supplementary material for: Integrated Transcriptome and Metabolomics to Reveal the Mechanism of Adipose Mesenchymal Stem Cells in Treating Liver Fibrosis
Source: Int J Mol Sci. 2023 Nov 8;24(22):16086. doi: 10.3390/ijms242216086 (PMC10671340; doi:10.3390/ijms242216086)
Supplement: Supplementary file 1 [file ijms-24-16086-s001.zip › Supplymentary S1.pdf]

Supplementary Table S1 86 Common metabolites detected in CG *vs* AG and AG *vs* SG

Name	VIP		Flod change		P-value	
	CCl4 vsCont	MSC vs CCl4	CCl4 vsCont	MSC vs CCl4	CCl4 vs Cont	MSC vs CCl4
Pc 40:8	2.822105816	2.914395406	0.466595328	2.206992485	1.81067E-08	1.09615E-07
N-(1.3-dihydroxyoctadeca-4.14-dien-2-yl)palmitamide	1.302602969	1.120001439	0.343679044	2.488300862	1.30563E-05	0.000696457
Sulfobacin b	3.279067407	2.392950457	0.190255171	3.206686303	3.8713E-05	0.000146029
3'-AMP	3.792924072	3.82129811	0.410915542	2.43526379	4.5051E-05	3.54254E-05
2-arachidonoyl-1-palmitoyl-sn-glycero-3-phosphoethanolamine	4.379610692	5.947356388	0.523457701	2.479335074	6.74124E-05	1.02361E-06
Pe(16:1e/14,15-epete)	2.25338666	1.73353827	0.258596574	2.602589514	0.000249671	5.5766E-05
Pantothenic acid	1.590269109	1.686220585	0.256132721	4.162027833	0.00060115	0.000276083
Perseitol	3.900944533	2.969983901	0.168929225	3.798648735	0.000668822	0.000212635
Pi 38:6	1.610419154	1.758757763	1.674308829	0.480628063	0.000733517	1.62482E-05
Pg 44:12	13.01163922	11.02044301	0.290725229	2.906943621	0.000921368	0.003833569
Pi 34:2	2.590062157	3.111002871	1.885149021	0.31757673	0.001037557	1.48774E-06
D(-)-beta-hydroxy butyric acid	1.361654061	2.193163446	0.38546552	6.163686647	0.001040585	0.008689147
Lithocholylglycine	1.167372411	1.132688218	0.121277961	7.720252925	0.001120794	0.000171174
Pg 40:8	3.922433465	4.458972189	1.599980883	0.477088743	0.002242185	0.000107546
12,13-dihydroxy-9z-octadecenoic acid	2.423675632	1.6122499	0.548317241	1.353009671	0.002249244	0.020415614
Pc(16:1e/8,9-epete)	2.048089829	1.488999859	0.507377963	1.442672793	0.002344008	0.002467593
Fahfa 36:3	4.147180201	3.362489754	1.936079189	0.609367051	0.002402051	0.004180275
Pg 34:3	1.313103767	1.28646576	0.568776517	1.740353244	0.002543022	0.004626494
Pg 40:7	3.287203639	2.320929638	2.791420667	0.571471367	0.003792399	0.037908881

Dihydrothymine	1.901872397	1.579217805	2.307926818	0.476818342	0.004728307	0.0209799
Pi 40:6	1.240791846	1.097471873	1.417066486	1.274786224	0.005364252	0.024293721
N-arachidonoyl-l-serine	1.191090302	1.018744659	0.195843061	3.825614259	0.009439979	0.000869279
Glycochenodeoxycholate	6.554647998	4.12177709	0.145747342	3.087267973	0.011255965	0.000308149
9-hydroxy-9-[[(e)-2-(hydroxymethyl)-3-[3-(hydroxymethyl)-6-propan-2-ylcyclohex-2-en-1-yl]prop-2-enoyl]oxymethyl]-1-oxo-6-propan-2-yl-3,5a,6,7,8,9a-hexahydro-2-benzoxepine-4-carboxylic acid	1.523383032	1.47677648	0.356993148	2.982558411	0.011406097	0.020493844
1-(9z,12z-octadecadienoyl)-2-hydroxy-sn-glycero-3-phosphoethanolamine	2.980123858	2.585587636	0.556483063	1.550799407	0.012534567	0.013747866
11,14,17-eicosatrienoic acid, (z,z,z)-	3.49687188	2.96569729	1.224380215	0.780492883	0.013075033	0.004756522
Cytidine monophosphate n-acetylneuraminic acid	1.440769074	1.525547689	2.089574315	0.366192259	0.013936468	0.003630513
Glycocholic acid	5.657230063	4.442708547	0.256739696	2.807868882	0.014194625	0.019892047
Pc(16:0e/8-hepe)	3.991654442	6.134756783	0.760247676	1.495557349	0.017668704	0.000490751
Lithosprmoside	1.184339828	1.27033622	0.555607287	1.752335615	0.024657998	0.013298112
Taurolithocholic acid sulfate	4.047223779	5.002852812	0.302462852	4.112150089	0.025053444	0.000344118
N-Acetylglucosamine 1-phosphate	1.99773785	1.641002776	1.624191519	0.662441082	0.025279328	0.011608967
1-stearoyl-2-arachidonoyl-sn-glycero-3-phospho-(1'-sn-glycerol)	1.650721904	1.377877107	2.085411695	0.5298635	0.028287118	0.041265267
1-octadecanoyl-sn-glycero-3-phospho-(1'-myo-inositol)	3.203127011	3.936173986	0.659600856	1.629169434	0.031964296	0.001227386
Pg 42:10	2.768057458	2.924882028	1.554079637	0.579191454	0.036661177	0.013553516
Linoleoylglycine	1.192867165	1.206164614	0.53587942	1.813023272	0.036869536	0.019700834

Indoxyl sulfate	5.807547849	6.731902805	2.671811488	0.114427652	0.04647939	0.007522515
Eplerenone hydroxy acid	2.12578183	2.86543607	0.374769384	3.812524015	0.047384812	0.003169966
2-piperidone	3.36503623	2.833862438	32.32059629	0.01718518	1.43708E-05	1.26117E-05
1,2-diarachidonoyl-sn-glycero-3-phosphocholine	6.555270448	5.951506845	0.494798087	2.173851709	1.50427E-05	1.09432E-06
1-Stearoyl-2-oleoyl-sn-glycerol 3-phosphocholine (SOPC)	3.23049229	3.160741319	0.464112972	2.490503627	1.52253E-05	1.1526E-06
Leu-Asp-Arg	2.62746961	2.014044775	0.173894815	5.100510945	4.72531E-05	8.95925E-06
2-Methylbutyroylcarnitine	6.680839135	5.631269519	4.082234695	0.225031088	5.47685E-05	3.88915E-05
Adenosine 2'-monophosphate	4.988609605	4.048064221	0.425741132	2.293268412	5.9868E-05	1.52508E-05
Perindopril	2.303340468	1.02290686	0.526545076	1.375578861	7.42724E-05	0.022370879
Glycodeoxycholic acid	4.87236966	3.246501439	0.160127239	4.523738579	0.000131186	0.000599315
2-oleoyl-1-palmitoyl-sn-glycero-3-phosphocholine	18.4940967	20.03072492	0.719978636	1.61913509	0.000148335	5.79708E-06
Glutathione, oxidized	7.026886824	6.077378893	1.652425726	0.548420323	0.000331417	0.000251391
4-oxoretinol	2.110470849	1.720661401	0.215449376	4.557312038	0.000351391	2.36246E-05
Thiazolidine-4-carboxylic acid	2.802130835	2.170019819	12.9934524	0.162762963	0.000415203	0.001123419
N-desbutylbupivacaine	2.115397007	1.273558885	0.143834635	3.958631033	0.000431658	5.54542E-05
G-guanidinobutyrate	8.854148144	7.9631935	4.626988671	0.10923599	0.000502806	0.000154065
1-o-hexadecyl-2-o-(5z,8z,11z,14z,17z-eicosapentaenoyl)-sn-glyceryl-3-phosphorylcholine	3.106880518	2.178861199	3.351567496	0.518953816	0.000534537	0.00553832
Caylin-1	1.342119729	1.097228365	1.50970595	0.661101223	0.00056706	0.001413845
Ammeline	1.46453244	1.324746244	5.203601285	0.079429152	0.000671108	0.000175008
Hirsutine	1.183988754	1.317897998	0.380496011	3.938796907	0.000822372	1.1226E-05

N-(4-fluorobenzoyl)-5-amino-1h-indole	10.27677192	6.021982519	0.15842535	3.803010393	0.001224873	0.000529038
Hexanoyl-l-carnitine	1.938095709	1.691713857	3.899110438	0.194223072	0.00139792	0.000607088
Nicotinamide	6.891306648	3.042421799	0.195498041	2.312259786	0.001675656	0.012475244
Thiamine	2.921399373	3.002520343	0.494297216	2.293825674	0.001711725	0.001805239
1,2-dilinoleoyl-sn-glycero-3-phosphoethanolamine	2.981204121	3.464273929	0.651901083	1.841491972	0.002054119	0.000101579
1-palmitoyl-2-arachidonoyl-sn-glycero-3-phosphoserine	1.384656152	1.177534154	0.451295663	2.332922246	0.002323442	0.000284513
Retinene	2.514982638	1.611576494	0.343137312	2.15589164	0.002491127	0.001062173
9-cis-Retinal	1.381341914	1.290344885	0.385365217	2.759105442	0.002534464	7.16721E-05
1-(1z-octadecenyl)-2-(5z,8z,11z,14z-eicosatetraenoyl)-sn-glycero-3-phosphocholine	3.444382848	2.472777753	3.27145847	0.502761733	0.002557151	0.012008062
1-(1z-octadecenyl)-sn-glycero-3-phosphocholine	3.910904434	2.943042262	8.606112884	0.27253385	0.002735659	0.008744942
13-cis-retinol	3.184854736	2.072976949	0.276060709	2.715522682	0.003031859	0.002410989
19,20-dihydroxy-4z,7z,10z,13z,16z-docosapentaenoic acid	1.63412436	1.343042115	0.412177427	2.347718518	0.003404789	0.000785629
Octanoylcarnitine	1.20706707	1.001921951	4.695807297	0.273035188	0.003411735	0.00577999
Glycocholate	1.787709172	1.378297273	0.137934172	5.862556704	0.003558905	0.003650952
2-docosahexaenoyl-1-palmitoyl-sn-glycero-3-phosphoethanolamine	4.057225089	3.235667826	1.280985224	0.815179964	0.003742363	0.011068506
4-[5-[[4-[5-[acetyl(hydroxy)amino]pentylamino]-4-oxobutanoyl]-hydroxyamino]pentylamino]-4-oxobutanoic acid	3.45820352	2.831163813	0.566445262	1.714109477	0.00596639	0.001864132

Adenosine 3'-monophosphate	2.375639393	2.172435929	0.571772339	1.65798459	0.006941827	0.004845895
Acetylcholine	7.700310638	5.21790018	3.370699471	0.483297273	0.007134062	0.032367712
1,2-dipentadecanoyl-sn-glycero-3-phosphocholine	1.216872605	1.149036701	1.400392869	0.685389222	0.010385168	0.004957173
1-(1z-hexadecenyl)-sn-glycero-3-phosphocholine	1.936420066	1.59682478	11.92107008	0.196679239	0.011780867	0.02276113
MG(18:2(9Z,12Z)/0:0/0:0)[rac]	1.657076103	1.584416003	1.523033212	0.558340875	0.012878158	0.001634527
N-alpha-acetyl-l-lysine	1.182669953	1.22952063	0.347083582	3.41907731	0.024078137	1.76526E-05
Ophthalmate	2.188952922	1.796555262	2.668121483	0.400449186	0.027311195	0.031845949
.beta.-estradiol 17-valerate	1.724187667	1.261247454	0.718436027	1.325952322	0.027923942	0.02710721
N-acetyl-d-glucosamine	1.535427866	1.862334718	0.59884663	2.075613096	0.028535177	0.000221949
L-propionylcarnitine	2.776960607	3.005487436	3.601621433	0.226762716	0.028759273	0.020491184
Hypoxanthine	14.97107181	16.62679345	0.698205333	1.694077665	0.034048859	0.000675594
(2r)-3-hydroxyisovaleroylcarnitine	1.547163273	1.267840289	2.37497807	0.468506102	0.035625558	0.035246105

Supplementary Table S2. Statistical comparison of clean data with data from a mouse reference genome

	Total reads	Mapped reads	Unique map reads	Multiple map reads	Reads map to '+'	Reads map to '-'
Cont-1	59724698	57387657(96.09%)	53242160(89.15%)	4145497(6.94%)	26595592(44.53%)	26646568(44.62%)
Cont-2	67220780	63641272(94.67%)	58956398(87.71%)	4684874(6.97%)	29406361(43.75%)	29550037(43.96%)
Cont-3	69286298	66412098(95.85%)	61853863(89.27%)	4558235(6.58%)	30852806(44.53%)	31001057(44.74%)
Cont-4	87237780	83877987(96.15%)	78269259(89.72%)	5608728(6.43%)	39053947(44.77%)	39215312(44.95%)
CCl_4-1	85929036	82980484(96.57%)	77271482(89.92%)	5709002(6.64%)	38591245(44.91%)	38680237(45.01%)
CCl_4-2	109982116	105569450(95.99%)	97082967(88.27%)	8486483(7.72%)	48472706(44.07%)	48610261(44.20%)
CCl_4-3	63693470	61037462(95.83%)	56178997(88.20%)	4858465(7.63%)	28018483(43.99%)	28160514(44.21%)

CCl_4-4	75013824	72304381(96.39%)	66730114(88.96%)	5574267(7.43%)	33328083(44.43%)	33402031(44.53%)
MSC-1	70780256	67587858(95.49%)	63196871(89.29%)	4390987(6.20%)	31544244(44.57%)	31652627(44.72%)
MSC-1	80346194	77151743(96.02%)	71901027(89.49%)	5250716(6.54%)	35918409(44.70%)	35982618(44.78%)
MSC-2	70048790	66554663(95.01%)	62277063(88.91%)	4277600(6.11%)	31114328(44.42%)	31162735(44.49%)
MSC-3	83654852	80440024(96.16%)	74696265(89.29%)	5743759(6.87%)	37273851(44.56%)	37422414(44.73%)
MSC-4	83654852	80440024(96.16%)	74696265(89.29%)	5743759(6.87%)	37273851(44.56%)	37422414(44.73%)
